# Integrated Expression Profiles Analysis Reveals Correlations Between the IL-33/ST2 Axis and CD8^+^ T Cells, Regulatory T Cells, and Myeloid-Derived Suppressor Cells in Soft Tissue Sarcoma

**DOI:** 10.3389/fimmu.2018.01179

**Published:** 2018-05-29

**Authors:** Huoying Chen, Yao Chen, Hongbo Liu, Yi Que, Xing Zhang, Fang Zheng

**Affiliations:** ^1^Department of Laboratory Medicine, The Second Affiliated Hospital of Guilin Medical University, Guilin, China; ^2^Melanoma and Sarcoma Medical Oncology Unit, State Key Laboratory of Oncology in South China, Collaborative Innovation Center for Cancer Medicine, Sun Yat-sen University Cancer Center, Guangzhou, China; ^3^Department of Immunology, School of Basic Medicine, Tongji Medical College, Huazhong University of Science and Technology, Wuhan, China; ^4^Department of Radiotherapy, Affiliated Hospital of Guilin Medical University, Guilin, China; ^5^Key Laboratory of Organ Transplantation, Ministry of Education, NHC Key Laboratory of Organ Transplantation, Chinese Academy of Medical Sciences, Wuhan, China

**Keywords:** IL-33, ST2, soft tissue sarcoma, IFN-γ, TGF-β1

## Abstract

Soft tissue sarcoma (STS) is a rare solid malignant cancer, and there are few effective treatment options for advanced disease. Cancer immunotherapy is a promising new strategy for STS treatment. IL-33 is a candidate cytokine for immunotherapy that can activate T lymphocytes and modulate antitumor immunity in some cancers. However, the expression and biological role of IL-33 in STS are poorly understood. In this study, we found that the expression of IL-33 and its receptor ST2 was decreased in STS using real-time PCR assays. By analyzing sarcoma data from The Cancer Genome Atlas, we found that higher transcriptional levels of IL-33 and ST2 were associated with a favorable outcome. There were positive correlations between the expression levels of ST2 and CD3E, CD4, CD8A, CD45RO, FOXP3, CD11B, CD33, and IFN-γ. Strong positive correlations between the expression of IFN-γ and CD3E and CD8A were also observed. Moreover, the expression levels of both IL-33 and ST2 were positively correlated with those of CD3E, CD8A, and chemokines that recruit CD8^+^ T cells, indicating that the IL-33/ST2 axis may play an important role in recruiting and promoting the immune response of type 1-polarized CD8^+^ T cells in STS. Meanwhile, we also found that the expression of IL-33 was negatively correlated with that of TGF-β1 and chemokines that recruit regulatory T cells (Tregs) and myeloid-derived suppressor cells (MDSCs), indicating that the IL-33/ST2 axis may also contribute to antagonizing Tregs, MDSCs, and TGF-β1-mediated immunosuppression in STS. The correlations between the IL-33/ST2 axis and CD8^+^ T cells and IFN-γ, as well as Tregs, MDSCs, and TGF-β1 were validated by additional analyses using three other independent GEO datasets of sarcoma. Our results implicate the possible role of the IL-33/ST2 axis in modulating antitumor immunity in STS. IL-33 may not only serve as a useful prognostic biomarker for STS but also as a potential therapeutic target for STS immunotherapy and worth further investigation.

## Introduction

Soft tissue sarcoma (STS) is a rare solid malignancy derived from mesenchymal tissues that accounts for approximately 1% of all cancers in adult patients (1). The pathogenesis of STS is poorly understood, and there are few effective treatment options for advanced disease. At present, despite the combination of surgery, chemotherapy, radiotherapy, and other systemic treatment, the overall 5-year survival rate of STS patients is only 50–60% (2). Recently, immunotherapy has emerged as a promising new treatment for cancer. For example, blockade of immune checkpoints has shown remarkable success in the treatment of melanoma, lung cancer, and colorectal cancer (3–5). Immunotherapy also offers new strategies for STS treatment. However, many mechanisms responsible for the failure of antitumor immunity, including active immunosuppression by the tumor microenvironment and insufficient immune stimulatory signals, have not yet been fully elucidated in STS, which limits the development of immunotherapy for STS.

IL-33 is a nuclear cytokine from the IL-1 cytokine family, and its role in immune moderation has been widely studied (6, 7). IL-33 is constitutively expressed in epithelial barrier tissues and lymphoid organs and functions as an alarmin (6, 8, 9). At the site of inflammation and damage, IL-33 is rapidly released from producing cells and activates the downstream NF-κB and MAPK pathways via a heteromeric receptor that consists of ST2 (also known as IL1RL1) and IL-1R accessory protein (IL-1RAcP) (10), thereby regulating the transcription of a variety of chemokines and cytokines and recruiting local immune cells to the site of inflammation and injury (9). IL-33 has been well established as a pleiotropic cytokine that regulates T helper 2 (Th2) cells, Th17/1 cells, and regulatory T cells (Tregs)-mediated immune responses (11–14). Recently, evidence has also shown that IL-33 can play an antitumor role by promoting the immune response of natural killer cells (NKs) and CD8^+^ T cells and enhancing IFN-γ production (15–17), which suggests that IL-33 is a potent cytokine for reversing the immunosuppressive tumor microenvironment and promoting antitumor immunity (16, 18). By analyzing publicly available tumor data from The Cancer Genome Atlas (TCGA), we found that IL-33 and ST2 mRNA is widely expressed in sarcoma, indicating that the IL-33/ST2 axis may play an important role in regulating antitumor immunity in STS. However, no reports currently define the role of the IL-33/ST2 axis in STS. Therefore, we analyzed the expression of IL-33/ST2 axis-related genes and clinical survival data of sarcoma from TCGA and GEO, hoping to provide clues as to whether IL-33 can modulate antitumor immunity and reverse the immunosuppressive tumor microenvironment in STS.

In the current study, we found that the mRNA expression of IL-33 and ST2 was decreased in STS. TCGA data analysis indicated that higher transcriptional levels of IL-33 and ST2 in STS were associated with a favorable outcome. By analyzing sarcoma data of TCGA and GEO, we found that the transcriptional levels of IL-33 and ST2 were positively correlated with those of CD3E, CD8A, IFN-γ, and chemokines that recruit CD8^+^ T cells, indicating that the IL-33/ST2 axis may play an important role in promoting the recruitment of CD8^+^ T cells and enhancing IFN-γ production in STS. We also found that the transcriptional level of IL-33 was negatively correlated with that of TGF-β1, an immunosuppressive cytokine, and chemokines that recruit Tregs and myeloid-derived suppressor cells (MDSCs), indicating that the IL-33/ST2 axis may also contribute to inhibiting the production of TGF-β1 and reducing the infiltration of Tregs and MDSCs in STS. Our results implicate the possible role of the IL-33/ST2 axis in the modulation of antitumor immunity in STS. IL-33 may serve as a useful prognostic biomarker for STS and a potential immunotherapeutic target for STS.

## Materials and Methods

### Human Sarcoma Specimens

A total of 18 pairs of sarcoma and adjacent tissue specimens used for real-time PCR assays of IL-33 and ST2 expression were collected from Sun Yat-sen University Cancer Center (SYSUCC), Guangzhou, China. Each biopsy specimen was immersed in RNAlater reagent overnight at 4°C and then preserved at −80°C until RNA extraction. Ethical approval was given by the Institutional Research Medical Ethics Committee of Sun Yat-sen University Cancer Center, and written informed consent was obtained from patients for the use of their clinical tissues in this study.

### RNA Extraction, Reverse Transcription, and Real-Time PCR

Total RNA was extracted from sarcoma specimens using TRIZOL reagent (Sigma-Aldrich, St. Louis, MO, USA) according to the manufacturer’s instructions. The RNA concentration and quantity were determined using a NanoDrop spectrophotometer (ND-1000, Thermo Scientific, USA). The first-strand cDNA was synthesized from 1 µg of total RNA using Superscript III Reverse Transcriptase (Invitrogen, Carlsbad, CA, USA). The expression of IL-33 and ST2 was detected using real-time PCR according to the protocol supplied by the manufacturer (Bio-Rad, CA, USA), and amplification was monitored with iQTM SYBR Green Supermix (Bio-Rad, CA, USA) according to the manufacturer’s instructions. The following primers were used for SYBR Green qPCR:

*IL-33*-forward: 5′-GTGACGGTGTTGATGGTAAGAT-3′
*IL-33*-reverse: 5′-AGCTCCACAGAGTGTTCCTTG-3′
*ST2*-forward: 5′-AGAAATCGTGTGTTTGCCTCA-3′
*ST2*-reverse: 5′-TCCAGTCCTATTGAATGTGGGA-3′
β*-actin*-forward: 5′-CGCGAGAAGATGACCCAGAT-3′
β*-actin*-reverse: 5′-GGGCATACCCCTCGTAGATG-3′

The expression data were normalized to the geometric mean of the housekeeping gene *β-actin* to control the variability in expression levels and calculated as 2^−ΔCT^ [ΔC_T_ = (C_T_ of gene) − (C_T_ of *β-actin*)], where C_T_ represents the threshold cycle for each transcript.

### TCGA Sarcoma Samples

Gene level 3 TCGA mRNA expression data were downloaded from the publicly accessible TCGA portal.^1^ Informed consent was provided by patients participating in TCGA program based on the guidelines from the TCGA Ethics, Law and Policy Group. The mRNA data were normalized by the RSEM algorithm and included 261 sarcoma patient samples. TCGA survival data with matched mRNA expression data from sarcoma were downloaded from OncoLnc.^2^ A heat map of the transcriptional expression data was generated with novel HemI software (Heatmap Illustrator, version 1.0) (19).

### GEO Data Series of Sarcoma

The datasets of transcriptome profiling by microarray were searched on GEO Profiles^3^ with the keywords “sarcoma *il33*” and limited the Organism to be “Homo sapiens.” Finally, three microarray datasets of sarcoma (GSE2719, GSE6481, and GSE 967) were screened and downloaded for analysis. Summary of the three selected GEO data series is shown in Table 1.

**Table 1:** Summary of the three GEO data series used in this study.

Data set	Microarray platform	Sarcoma samples	Publication
GSE2719	GPL96	39	*Cancer Res* 2005; PMID: 5994966
			*J Surg Res* 2006; PMID: 16603191
GSE6481	GPL96	105	*Mod Pathol* 2007; PMID: 17464315
GSE967	GPL91	23	*Int J Cancer* 2004; PMID: 15146558

### Statistics

All of the statistical analyses were performed using SPSS standard version 16.0 (SPSS Inc., Chicago, IL, USA) and GraphPad Prism version 5.0 (GraphPad Software, San Diego, CA, USA). A paired *t*-test was used when two paired measurements were analyzed. The correlation between gene expression levels was analyzed by Pearson correlation test. Survival analysis was performed by Kaplan–Meier and Log-rank test. *P*-values less than 0.05 were considered statistically significant.

## Results

### Higher Expression Levels of IL-33 and ST2 Are Associated With a Favorable Prognosis in STS

**Figure 1:**
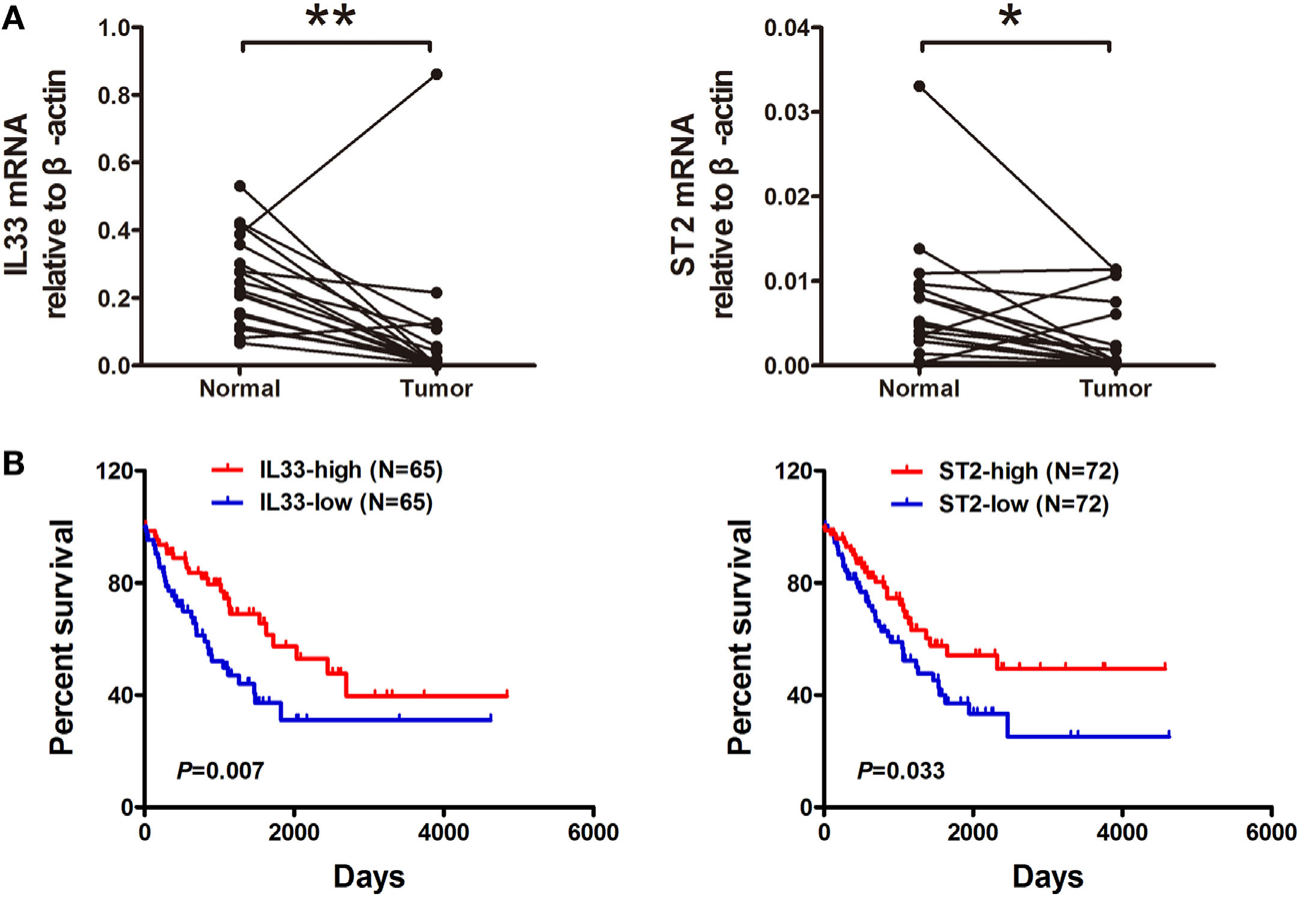
Expression and prognostic significance of IL-33 and ST2 in soft tissue sarcoma. **(A)** The expression of IL-33 and ST2 in tumor tissues was lower than those in adjacent normal tissues. A total of 18 pairs of sarcoma and adjacent normal tissue specimens were collected and used for real-time PCR assays for IL-33 and ST2 expression. The expression data were normalized to the geometric mean of the housekeeping gene β-actin. *P < 0.05, **P < 0.01 using paired *t*-test. **(B)** Higher transcriptional levels of IL-33 and ST2 were associated with a better outcome. Transcriptional sequencing and survival data from patients with soft tissue sarcoma were obtained from the publicly accessible The Cancer Genome Atlas portal, and overall survival analysis was evaluated by Kaplan–Meier and Log-rank test.

To evaluate the expression of IL-33 and ST2 in STS, we examined 18 pairs of sarcoma and adjacent normal tissue specimens for IL-33 and ST2 expression using real-time PCR assays. We found that the expression levels of IL-33 and ST2 were decreased in tumor tissues compared with adjacent normal tissues (Figure 1A). Furthermore, we analyzed transcriptome sequencing and survival data from sarcoma from the TCGA and found that both IL-33 and ST2 expression were associated with the prognosis of sarcomas. Patients with higher transcriptional levels of IL-33 and ST2 have a more favorable prognosis (Figure 1B). These results indicate that IL-33 and ST2 are involved in the progression of STS and may serve as useful prognostic biomarkers for STS.

### IL-33 and ST2 Are Positively Correlated With the Expression of Different Immune Cell Subpopulation-Specific Genes in STS

**Figure 2:**
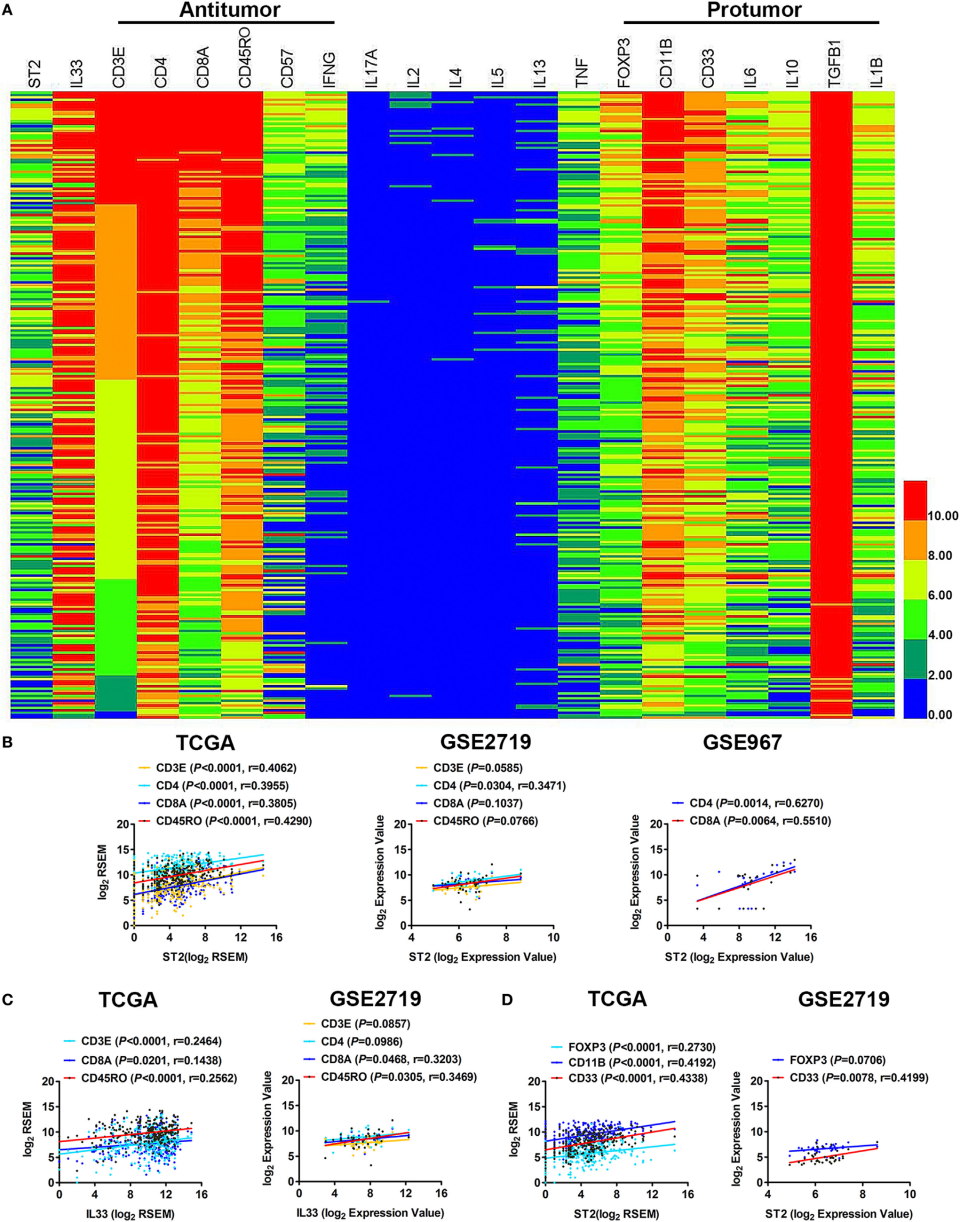
Correlation between the expression of ST2 and different immune cell subpopulation-specific genes in soft tissue sarcoma (STS). **(A)** The transcriptional expression of antitumor and pro-tumor immune components in the tumor microenvironment of STS. The transcriptome sequencing data (sample number = 261) were obtained from the publicly accessible The Cancer Genome Atlas (TCGA) portal, and the heat map was generated with novel HemI software (Heatmap Illustrator, version 1.0). CD3E and CD4 were used to label T cells; CD8A labeled cytotoxic T lymphocytes; CD45RO labeled memory T cells; CD57 labeled activated T cells and NK cells; FOXP3 labeled Tregs; and CD11B and CD33 labeled myeloid-derived suppressor cells. These data are in descending order according to the level of CD3E expression. Highly expressed samples are in red, and samples with lower expression are in blue. **(B)** Positive correlations between the transcriptional levels of ST2 and CD3E, CD4, CD8A, and CD45RO by analyzing sarcoma data of TCGA, GSE2719 (sample number = 39) and GSE967 (sample number = 23). **(C)** Positive correlations between the transcriptional levels of IL-33 and CD3E, CD8A, and CD45RO. **(D)** Positive correlations between the transcriptional levels of ST2 and FOXP3, CD11B, and CD33. The correlation between gene expression levels was analyzed by Pearson correlation test.

We analyzed the immunological components in the tumor microenvironment of STS and found that the components of the antitumor immune response in STS were mainly CD3E/CD4/CD8A-labeled T cells, CD57-labeled NK cells, and CD45RO-labeled memory T cells that can secrete IFN-γ. The components that inhibited the immune response were primarily FOXP3-labeled Tregs and CD11B/CD33-labeled MDSCs, as well as IL-10, IL-6, TGF-β1, and other immunosuppressive cytokines (Figure 2A). In particular, the expression of TGF-β1 was very high. It is likely that TGF-β1 is the major immunosuppressive cytokine in the tumor microenvironment of STS. Meanwhile, the expression levels of IL-17A, IL-2, and the Th2 cytokines IL-4, IL-5, and IL-13 were almost undetectable (Figure 2A), indicating that the Th17 and Th2 immune responses were almost ineffective in STS. It has been reported that Th1, CD8^+^ T cells, NKs, Tregs, and MDSCs can express ST2 (7, 9, 16, 20), and IL-33 can directly regulate the immune function of these cells through ST2. To determine in which of the above cells the IL-33/ST2 axis may play a role in STS, we performed a series of correlation analyses. By analyzing TCGA data, we found that there were positive correlations between the expression of ST2 and CD3E, CD4, CD8A, and CD45RO (Figure 2B). Weak correlations between the expression of IL-33 and CD3E, CD8A, and CD45RO were also observed (Figure 2C). There were also positive correlations between the expression of ST2 and FOXP3, CD11B, and CD33 (Figure 2D). The correlations between the expression of ST2 and CD4, CD8A, and CD33, and between IL-33 and CD8A and CD45RO were then validated by analysis using GSE2719 or GSE 967 GEO datasets of sarcoma (Figures 2B–D). However, there was no correlation between the expression of ST2 and CD57 or between IL-33 and CD57 (data not shown). Taken together, these results indicate that the IL-33/ST2 axis may regulate the immune function of Th1, CD8^+^ T cells, and memory T cells, as well as Tregs and MDSCs in the tumor microenvironment of STS.

### ST2 Is Positively Correlated With the Expression of IFN-γ in STS

**Figure 3:**
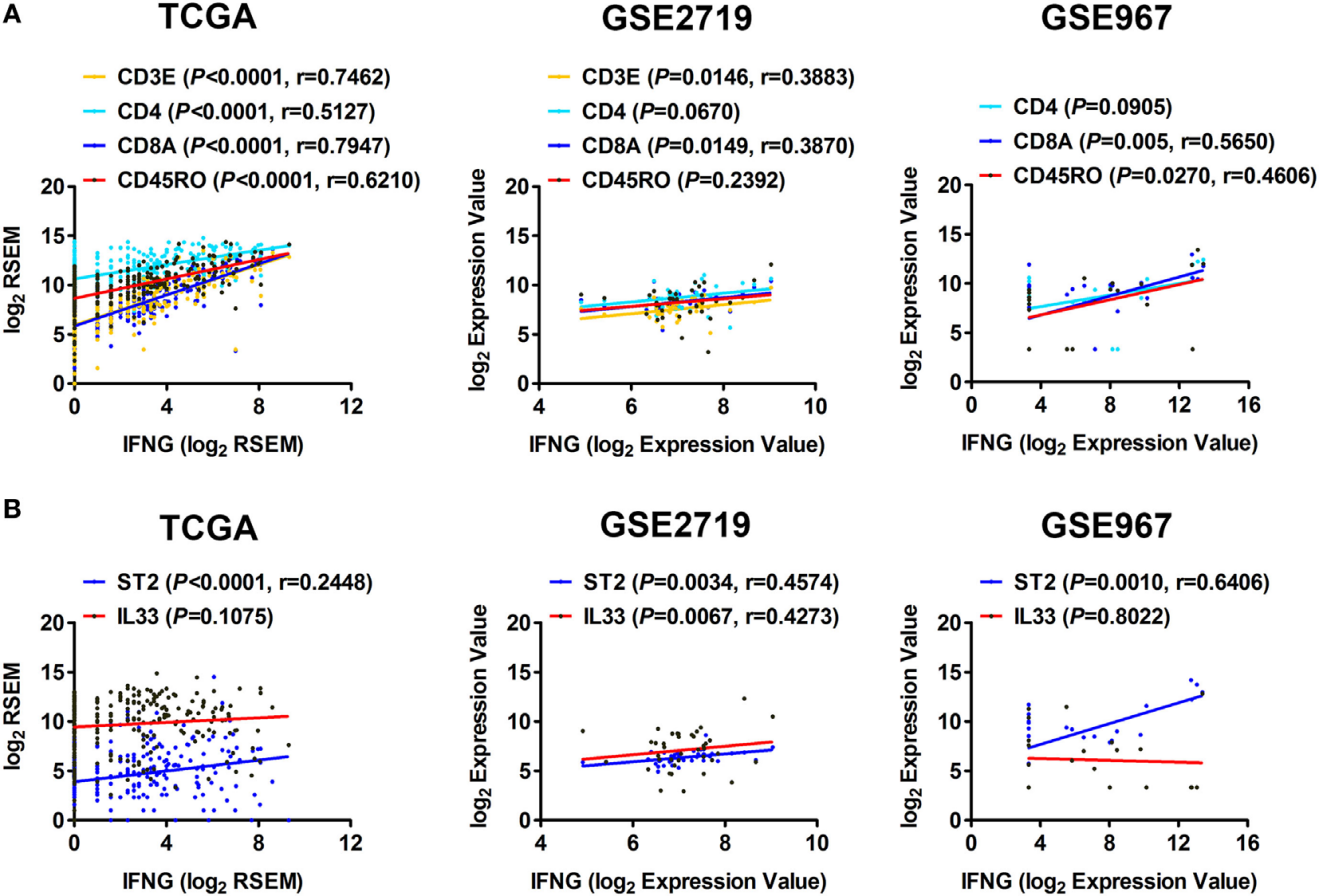
Correlation between the expression of ST2 and IFN-γ in soft tissue sarcoma. **(A)** Positive correlations between the transcriptional levels of IFN-γ and CD3E, CD4, CD8A, and CD45RO by analyzing sarcoma data of The Cancer Genome Atlas, GSE2719, and GSE967. **(B)** Positive correlations between the transcriptional levels of IFN-γ and ST2, IL-33. The correlation between gene expression levels was analyzed by Pearson correlation test.

IFN-γ is an important cytokine in antitumor responses (16). We further explored whether the IL-33/ST2 axis is associated with the production of IFN-γ in STS. Correlation analyses using sarcoma data of TCGA showed that the expression of IFN-γ was positively correlated with that of CD4 and CD45RO, but strong positive correlations were observed with CD3E and CD8A expression (Figure 3A). This suggests that CD3E/CD8A-labeled T cells may be the major cells that secrete IFN-γ in STS. The correlations between the expression of IFN-γ and CD3E, CD8A, and CD45RO were then validated by analysis using GSE2719 or GSE967 datasets of sarcoma (Figure 3A). Moreover, we found that the expression of ST2 was positively correlated with that of IFN-γ (Figure 3B). Positive correlation between the expression of IFN-γ and IL-33 was also observed by analysis using GSE2719 dataset (Figure 3B). Combined with the results indicating that the expression of ST2 is positively correlated with that of CD3E, CD4, CD8A, and CD45RO (Figure 2A), it is easy to deduce that the IL-33/ST2 axis may promote T cells, especially CD8^+^ T cells, to produce IFN-γ in STS.

### IL-33 and ST2 Are Positively Correlated With the Expression of Chemokines That Recruit CD8^+^ T Cells in STS

**Figure 4:**
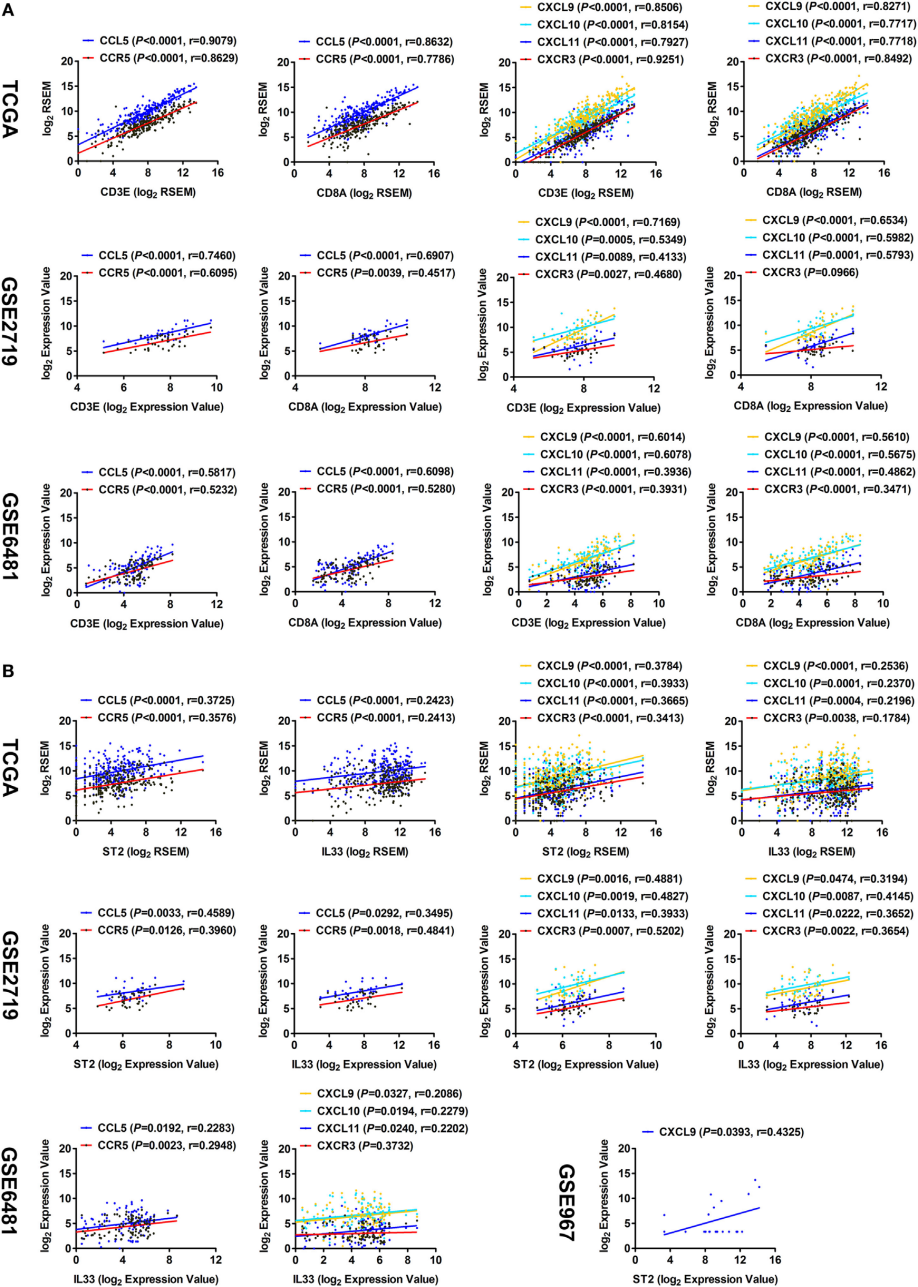
Correlation between the expression of ST2/IL-33 and chemokines that recruit CD8^+^ T cells in soft tissue sarcoma. **(A)** Positive correlations between the transcriptional levels of CD3E/CD8A and CCL5 and its receptor CCR5, and between CD3E/CD8A and CXCL9/10/11 and their receptor CXCR3 by analyzing sarcoma data of The Cancer Genome Atlas (TCGA), GSE2719, and GSE6481 (sample number = 105). **(B)** Positive correlations between the transcriptional levels of ST2/IL-33 and CCL5 and its receptor CCR5, and between ST2/IL-33 and CXCL9/10/11 and their receptor CXCR3 by analyzing sarcoma data of TCGA, GSE2719, GSE6481, and GSE967. The correlation between gene expression levels was analyzed by Pearson correlation test.

It has been reported that the chemokine CCL5 and its receptor CCR5, as well as CXCL9, CXCL10, CXCL11, and their receptor CXCR3, are involved in recruiting CD8^+^ T cells in the tumor microenvironment (21–23). Here, correlation analyses of TCGA data showed that the expression levels of CCL5 and CCR5, as well as CXCL9, CXCL10, CXCL11, and CXCR3, had strong correlations with those of CD3E and CD8A (Figure 4A). The correlations were then validated by analysis using GSE2719 and GSE6481 datasets of sarcoma (Figure 4A). Moreover, the expression levels of both ST2 and IL-33 were found to be positively correlated with those of CCL5, CCR5, CXCL9, CXCL10, CXCL11, and CXCR3 (Figure 4B). These results suggest that the IL-33/ST2 axis may recruit CD8^+^ T cells into cancer lesions by promoting the release of multiple chemokines in the tumor microenvironment of STS.

### IL-33 Is Negatively Correlated With the Expression of TGF-β1 and Chemokines That Recruit Tregs and MDSCs in STS

**Figure 5:**
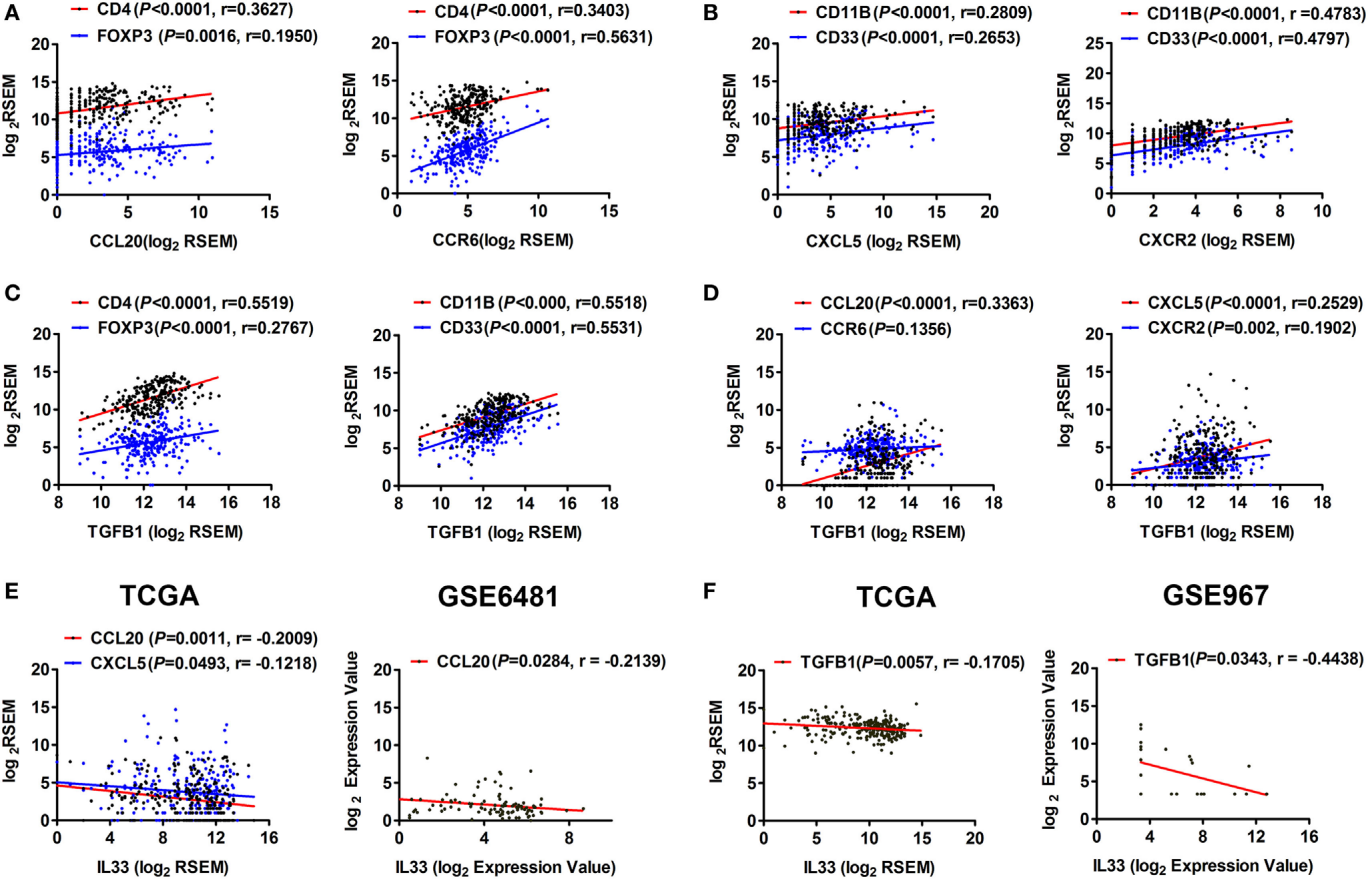
Correlation between the expression of IL-33 and TGF-β1, chemokines that recruit Tregs and myeloid-derived suppressor cells in soft tissue sarcoma. **(A)** Positive correlations between the transcriptional levels of CCL20 and its receptor CCR6 and CD4/FOXP3. **(B)** Positive correlations between the transcriptional levels of CXCL5 and its receptor CXCR2 and CD11B/CD33. **(C)** Positive correlations between the transcriptional levels of TGFB1 and CD4/FOXP3 and CD11B/CD33. **(D)** Positive correlations between the transcriptional levels of TGFB1 and CCL20, CXCL5, and CXCR2. Above analyses were performed using data from The Cancer Genome Atlas (TCGA). **(E)** Negative correlations between the transcriptional levels of IL-33 and the chemokines CCL20 and CXCL5 by analyzing sarcoma data of TCGA and GSE6481. **(F)** Negative correlation between the transcriptional levels of IL-33 and TGFB1 by analyzing sarcoma data of TCGA and GSE967. The correlation between gene expression levels was analyzed by Pearson correlation test.

To determine whether the IL-33/ST2 axis is also involved in the regulation of immunosuppressive cells and cytokines, we performed a series of correlation analyses. It has been reported that the chemokine CCL20 and its receptor CCR6 recruit Tregs into the inflammation site (24, 25), while CXCL5 and its receptor CXCR2 recruit MDSCs and promote TGF-β1 secretion (26, 27). Here, by analyzing TCGA data, we found that the expression levels of CCL20 and CCR6 were positively correlated with those of CD4 and FOXP3 (Figure 5A), while CXCL5 and CXCR2 levels were positively correlated with those of CD11B and CD33 in STS (Figure 5B). As shown in Figure 2A, TGF-β1 is highly expressed in STS. We found that there was a weak positive correlation between the expression of TGFB1 and FOXP3 but obvious positive correlations between the expression of TGFB1 and CD11B and CD33 (Figure 5C), indicating that CD11B/CD33-expressing MDSCs may be the main cells, which secrete TGF-β1. Furthermore, positive correlations were observed between the expression of TGFB1 and CCL20, CXCL5, and CXCR2 (Figure 5D). At the same time, we found that IL-33 was negatively correlated with the expression of the chemokines CCL20 and CXCL5, as well as with TGF-β1 (Figures 5E,F). The negative correlations between the expression of IL-33 and CCL20 and TGFB1 were then validated by analysis using GSE6481 or GSE967 datasets of sarcoma (Figures 5E,F). These results suggest that the IL-33/ST2 axis may also inhibit the production of TGF-β1 and reduce the infiltration of Tregs and MDSCs by inhibiting the expression of chemokines such as CCL20 and CXCL5 in the tumor microenvironment of STS, thus contribute to antagonizing Tregs, MDSCs, and TGF-β1-mediated immunosuppression.

## Discussion

Cancer immunotherapy opens up new avenues of treatment for many types of cancers, including STS. At present, a number of immunotherapy clinical trials for STS are ongoing, including treatment with anti-PD-1 antibody (28) and adoptive transfer of T cells targeting NY-ESO-1 antigen (29). However, despite some achievements in cancer immunotherapy, patients with STS have a low response rate. In a Phase I clinical trial using a SYT-SSX peptide vaccine in patients with synovial sarcoma, peptide-specific CD8 cytotoxic T lymphocytes were successfully induced in 4 of 6 patients, but suppression of tumor progression only occurred in one patient (30). In another small clinical trial investigating the clinical activity of the anti-CTLA4 antibody Ipilimumab in patients with synovial sarcoma expressing NY-ESO-1 antigens, only 1 of the 6 patients showed remission (31).

The major obstacle to immunotherapy is immunosuppression in the tumor microenvironment. Immunosuppression includes the accumulation of Tregs and MDSCs as well as the production of various immunosuppressive cytokines in the tumor microenvironment (32). The type I immune responses mediated by Th1, CD8^+^ T, NK, NKT, and γδ T cells that produce IFN-γ are considered to be the basis of antitumor immune responses (33). However, immunosuppression in the tumor microenvironment results in these immune cells being in a dysfunctional immune state, which eventually leads to the immune escape of tumor cells (32, 34). Therefore, reversing immunosuppression in the tumor microenvironment is a crucial step toward the success of cancer immunotherapy.

Cytokines have been shown to activate immune responses and have antitumor effects. A variety of cytokines have been approved as drugs for the treatment of cancer patients, including IL-2, IFN-γ, and GM-CSF (35, 36). Recently, studies have shown that IL-33 also has antitumor activity in some cancers, such as melanoma and breast cancer (15–17). IL-33 could be released by damaged or dead tumor cells and act on immune cells that express ST2 in a paracrine or autocrine manner (37). In this study, we found that the expression of IL-33 and ST2 were decreased in STS. Furthermore, we found that both IL-33 and ST2 expression were associated with the prognosis of sarcomas; patients with higher transcriptional levels of IL-33 and ST2 have a more favorable prognosis. These results indicate that IL-33 may be released from sarcoma cells and regulate the antitumor immunity during the progression of STS.

It has been reported that the antitumor effect of IL-33 is mainly achieved by promoting the production of IFN-γ by NK and CD8^+^ T cells (15–17). In addition, IL-33 can also promote NK and NKT cells to produce IFN-γ (13, 38). IL-33 is also able to recruit large amounts of type 2 innate lymphoid cells to the tumor lesions and inhibit tumor growth (39). Thus, IL-33 is a potent pleiotropic cytokine that reverses immunosuppression in the tumor microenvironment. Here, we found that the expression level of IFN-γ was positively correlated with those of CD4 and CD45RO, but strong positive correlations were observed with the levels of CD3E and CD8A, indicating that CD3E/CD8A-labeled T cells may be the major cells that secrete IFN-γ in STS. Additionally, there was a positive correlation between the expression of ST2 and IFN-γ. Combined with the results indicating that the expression levels of both ST2 and IL-33 were positively correlated with those of CD3E, CD8A, and CD45RO, it is easy to deduce that the IL-33/ST2 axis may promote T cells, especially CD8^+^ T cells, to produce IFN-γ in STS. Furthermore, both the expression of ST2 and IL-33 were found to be positively correlated with that of CCL5 and its receptor CCR5, as well as with the expression of CXCL9, CXCL10, CXCL11, and their receptor CXCR3. All of these chemokines have been reported to recruit CD8^+^ T cells to the site of inflammation and participate in antitumor immune responses (21–23). Taken together, these results suggest that the IL-33/ST2 axis may enhance the antitumor immunity by promoting the recruitment recruitment of type 1-polarized CD8+ T cells into tumor lesions in STS.

However, it has also been reported that IL-33 can promote the accumulation of ST2^+^ Tregs in tumor lesions and exhibits an immunosuppressive effect (16); IL-33 can also promote MDSCs to accumulate in the tumor and secrete a large amount of immunosuppressive cytokines, such as TGF-β1, resulting in tumor metastasis (40). In this study, we found that there were positive correlations between the expression of ST2 and FOXP3, CD11B, and CD33, indicating that the IL-33/ST2 axis may also regulate the function of Tregs and MDSCs in the tumor microenvironment of STS. TGF-β1 is highly expressed in STS; it is likely that TGF-β1 is the major immunosuppressive cytokine in the STS tumor microenvironment. We also found that there were obvious positive correlations between the expression levels of TGFB1 and CD11B and CD33, indicating that CD11B/CD33-labeled MDSCs may be the main cells that secrete TGF-β1 in STS. It has been reported that the CCL20-CCR6 axis recruits Tregs (24, 25) and the CXCL5-CXCR2 axis can recruit MDSCs (26, 27) into the inflammation site. Here, we found that the expression levels of CCL20 and CCR6 were positively correlated with those of FOXP3, while CXCL5 and CXCR2 were positively correlated with CD11B and CD33. Furthermore, positive correlations were observed between the expression levels of TGFB1 and CCL20 and CXCL5. The above results indicate that TGF-β1 may play a major immunosuppressive role in STS. However, we found that IL-33 was negatively correlated with the expression of the chemokines CCL20 and CXCL5, as well as with TGF-β1, in STS, suggesting that the IL-33/ST2 axis may reverse immunosuppression mainly by reducing the infiltration of Tregs and MDSCs and inhibiting the production of TGF-β1 in the STS tumor microenvironment. The regulatory effect of IL-33 on TGF-β1 is controversial. Some studies have shown that IL-33 induces the production of TGF-β by eosinophils and M2 macrophages (41, 42), and some studies have reported that treatment with anti-IL-33 antibody or sST2 in allergic asthma did not change the level of TGF-β1 (43). Whether IL-33 inhibits TGF-β1 production in the STS microenvironment and the mechanism involved remain to be confirmed.

**Figure 6:**
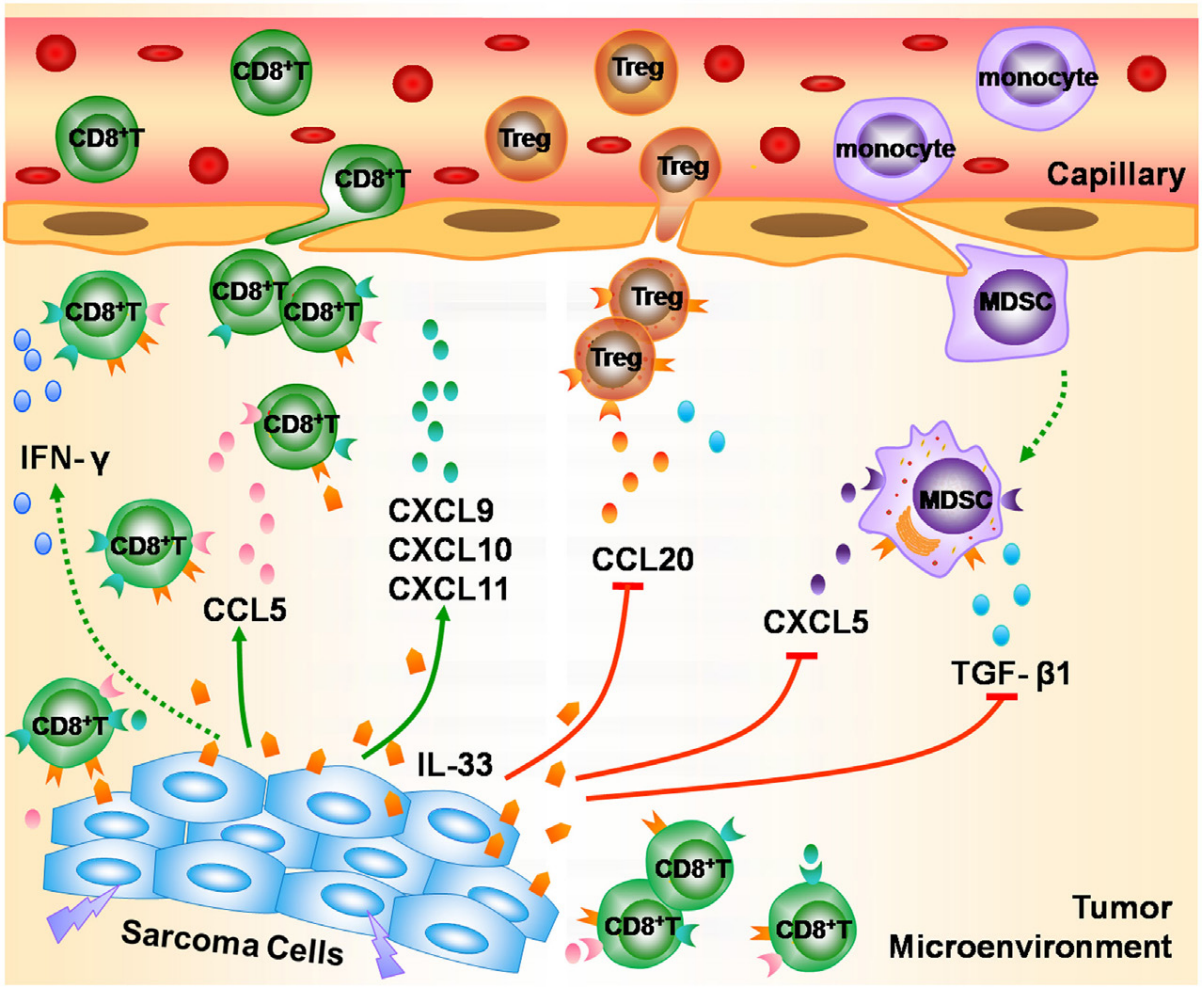
The possible role of the IL-33/ST2 axis in modulating antitumor immunity in soft tissue sarcoma (STS). During the development of STS, IL-33 is released from damaged or dead sarcoma cells into the tumor microenvironment, and enhances the recruitment of CD8^+^ T cells into tumor lesion by promoting the release of multiple chemokines such as CCL5, CXCL9, CXCL10, and CXCL11, and promotes CD8^+^ T cells to produce IFN-γ, thereby playing an important role in the modulation of antitumor immunity in STS. Meanwhile, IL-33/ST2 axis reduces the infiltration of Tregs and myeloid-derived suppressor cells *via* inhibiting the production of chemokines CCL20 and CXCL5, and suppresses the secretion of TGF-β1, and thus contributes to antagonizing the immunosuppression in STS.

In summary, we found that the mRNA expression of IL-33 and ST2 was decreased in STS. TCGA data analysis indicated that higher transcriptional levels of IL-33 and ST2 in STS were associated with a favorable outcome. Integrated analysis of TCGA and GEO sarcoma datasets implicated the possible role of the IL-33/ST2 axis in STS. IL-33/ST2 may play an important role in the modulation of antitumor immunity in STS by promoting the recruitment of CD8^+^ T cells and enhancing IFN-γ production, as well as by antagonizing Tregs, MDSCs, and TGF-β1-mediated immunosuppression (Figure 6). Our study suggests that IL-33 may not only serve as a useful prognostic biomarker for STS but also as a potential immunotherapeutic target for STS. However, further *in vivo* and *in vitro* experiments are required to validate the possible antitumor effect of the IL-33/ST2 axis on STS.

## Ethics Statement

This study was carried out in accordance with the recommendations of ICH-QCP guidelines, Institutional Research Medical Ethics Committee of Sun Yat-sen University Cancer Center. The protocal was approved by the Institutional Research Medical Ethics Committee of Sun Yat-sen University Cancer Center. The written informed consent was obtained from patients for the use of their clinical tissues in this study.

## Author Contributions

Initiation and study design: FZ; clinical samples contribution: HC, YQ, and XZ; performed experiments: HC; statistical analyses: HC, HL, and YC; supervision of research: FZ; writing of the first draft of the manuscript: HC and YC. All authors contributed to the writing and editing of the current manuscript and approved the final manuscript.

## Conflict of Interest Statement

The authors declare that the research was conducted in the absence of any commercial or financial relationships that could be construed as a potential conflict of interest.

## Footnotes

^1^https://portal.gdc.cancer.gov/ (Accessed: March 24, 2016).

^2^http://www.oncolnc.org/ (Accessed: November 30, 2016).

^3^https://www.ncbi.nlm.nih.gov/geoprofiles/ (Accessed: February 10, 2017).
